# Exploratory metabolomic analysis for identifying systemic signatures of *Helicobacter pylori* infection in children

**DOI:** 10.3389/fimmu.2026.1826604

**Published:** 2026-06-16

**Authors:** Weronika Gonciarz, Lucyna Kozlowska, Marta Stelmasiak, Magdalena Wozniczka, Magdalena Chmiela

**Affiliations:** 1 Department of Immunology and Infectious Biology, Faculty of Biology and Environmental Protection, University of Lodz, Lodz, Poland; 2 Department of Dietetics, Institute of Human Nutrition Sciences, Warsaw University of Life Sciences, Warsaw, Poland; 3 Department of Physical and Biocoordination Chemistry, Faculty of Pharmacy, Medical University of Lodz, Lodz, Poland

**Keywords:** children, diagnostic signatures, *H. pylori* infection, serum samples, untargeted metabolomic profile

## Abstract

**Background:**

Infections caused by the Gram-negative bacteria *Helicobacter pylori* (*H. pylori*) can result in gastritis, gastric or duodenal ulcers, and gastric cancer in humans. Examining quantitative changes in soluble biomarkers linked to *H. pylori* infection offers a promising approach to monitor the infection’s progression, inflammatory response, and systemic effects.

**Aim:**

This exploratory study aimed to analyze metabolomic biomarkers in the sera from children with dyspeptic symptoms infected with *H. pylori* and in control group of healthy children not exposed to *H. pylori*.

**Materials and methods:**

Biological samples: sera from 32 *H. pylori*-infected children – Hp (+) (female and male) with gastrointestinal symptoms (GIs) under the pediatric gastroenterological medical care; sera from 32 *H. pylori* uninfected children – Hp(-) (female and male) without GIs under general medical care. The *H. pylori* status in Hp(+) children was confirmed by ^13^C urea breath testing, the presence of serum anti-*H. pylori* IgG antibodies and gastroscopy-based tests (rapid urease test, histological examination of gastric tissue specimens for the presence of Helicobacter-like organisms-HLO and inflammation) while Hp(-) group was selected based on negative result of ^13^C urea breath test and the lack of serum anti-*H. pylori* IgG antibodies. Metabolomic profiling was performed using UPLC-QTOF/MS methods. Biomarkers significantly associated with *H. pylori* infection were identified using volcano plots and ROC analysis.

**Results:**

This exploratory study found 7 metabolites differentiating the serum samples of Hp(+) from Hp(-) children: carboxyethyl lysine - CEL (HMDB29447), gamma-Glutamylleucine (HMDB0011171), 13-HOTrE(y) hydroxylated and oxidized derivative of the omega-6 fatty acid arachidonic acid (HMDB0341541), 13-HODE (HMDB0004667), lauroylcarnitine (HMDB0002250), vitamin A (HMDB0000305) and 19_norandrosterone (HMDB0002697). These metabolites are associated with immune regulation, energy metabolism, lipid/fatty acid metabolism, lipid peroxidation, oxidative stress, and cell signaling, which may be linked with the pathogenesis of *H. pylori* infection in humans. However, this hypothesis needs to be confirmed based on direct immune measurements and longitudinal clinical outcomes.

**Conclusions:**

This exploratory study delivered preliminary results on serum metabolomic profiling indicating differences between metabolites present in serum samples of *H. pylori*-infected and uninfected children. Further study is required for validation of proposed methodology and connecting the selected metabolites with *H. pylori* infection.

## 1 Introduction

*H. pylori*, a Gram-negative spiral-shaped rods described in 1983, colonize gastric epithelium approximately half of the world’s population (1). If not eradicated, these bacteria can persist for life. Although in about 80% of infected individuals, there are no specific symptoms, the infection may initiate deleterious effects in the gastric mucosa. In the stomach, *H. pylori* trigger a significant inflammatory response that can lead to conditions such as chronic gastritis, gastric and duodenal ulcers, and malignancies such as mucosa associated lymphoid tissue (MALT) lymphoma or gastric cancer (2–6). The infection’s progression depends on *H. pylori* virulence factors, the host’s susceptibility, and socio-economic conditions (7). Chronic character of *H. pylori* infections results from ineffective humoral and cellular immune responses against these microorganisms, whose components may interfere with the activity of immunocompetent cells (8). Persistent *H. pylori* infections, particularly those which are initiated by *H. pylori* strains producing cytotoxin associated gene A (CagA) protein along with a heightened local inflammatory response in the stomach, have been suggested as a risk factor of systemic inflammation and development of extragastric diseases such as immune thrombocytopenic purpura, iron deficiency anemia, and vitamin B12 deficiency (9–11). In other diseases, including cardiovascular disorders, diabetes mellitus, dermatological conditions, neurological disorders, and lung cancer, the role of *H. pylori* infection is also considered (12–16). The link between *H. pylori* infections and growth retardation in children due to iron deficiency or antigenic mimicry between *H. pylori* compounds and appetite-regulating peptides, thrombocyte proteins, or through the modulation of ghrelin and leptin secretion has been suggested (17–21). In children, symptoms of gastritis can include nausea, vomiting, and abdominal pain while children with peptic ulcer may additionally experience gastric bleeding, and due to this blood enriched stool. In younger children, symptoms might be less obvious, making diagnosis more challenging. During cyclic Maastricht meetings, the European Consensus Group (ECG) recommended using ^13^C urea breath testing and histological examination of gastric tissue samples for the detection of Helicobacter-like organisms and inflammation as reference diagnostic methods (22, 23). Testing stool samples for *H. pylori* antigens was also recommended, especially in fully symptomatic adult and pediatric patients, however with some limitations (24).

Recently, diagnostic recommendations have been rated by the European and North American Societies of Pediatric Gastroenterology, Hepatology and Nutrition experts according to PICO (patient population, intervention, comparator, and outcome) questions which were voted by the group (25). Recommendations were formulated using the Evidence to Decision framework. Invasive methods based on rapid urease test, histopathological examination and bacterial culture from gastric tissue followed by assessment of antibiotic resistance for selection of eradication therapy remain a gold standard. Molecular methods, which were proposed earlier for diagnosis of *H. pylori* in adults (26) are not included in this standard, however, they are acceptable both for detection of infection and assessment of antibiotic resistance using gastric biopsy specimens. Non-invasive tests, including stool antigen test and serological test, which allow detection of anti-*H. pylori* antibodies in serum samples can be used particularly as a screening method for children with history of gastric cancer in a first‐degree relative. Raso et al., confirmed that stool antigen test and urea breath test demonstrate high sensitivity, thus in children without alarm symptoms these tests may exclude *H. pylori* infection facilitating avoiding endoscopy (27). Invasive and noninvasive diagnostic tests have advantages and limitations depending on the clinical setting and the child’s age. Guidelines recommend not performing diagnostic testing in children with chronic or recurrent abdominal symptoms or cases of a family history of severe *H. pylori-*related disease, which often cause clinical dilemmas for diagnosis and treatment of *H. pylori* infection (28). Testing for *H. pylori* in chronic immune thrombocytopenic purpura, inflammatory bowel disease, celiac disease, or eosinophilic esophagitis is not recommended. Treatment is based on antibiotic sensitivity testing, and therapeutic protocols based on clarithromycin should be avoided.

Although the above recommended methods are sufficiently sensitive and specific for detecting *H. pylori* infection, they do not enable distinguishing systemic biomarkers reflecting *H. pylori-*related deleterious effects in children, including metabolic changes that may be related to delayed growth. Identifying metabolic markers that fluctuate during *H. pylori* infection may potentially facilitate selection of those which are related to *H. pylori-*driven systemic effects. Untargeted metabolomics is a common experimental biological technique that enables the analysis of metabolic responses in individual organisms or populations to drug treatments. By combining analytical chemistry methods with knowledge of biological processes, it is possible to identify and quantify cell, tissue, and body fluid metabolites (blood, plasma, serum, urine) or stool. It helps understand the host-pathogen interactions, detect changes reflecting disease and select disease related markers, ultimately enhancing the effectiveness of medical care for patients (29). Since the individual metabolic profile is influenced by both genetic and environmental factors, metabolomics may help to develop personalized treatment (30). For example, in biomedicine, methods such as nuclear magnetic resonance (NMR), gas chromatography-mass spectrometry (GC–MS), flow injection analysis-mass spectrometry (FIA-MS), and liquid chromatography-mass spectrometry (LC/MS) have been effectively used to analyze the metabolite profiles of obese patients, diabetes patients, as well as individuals with cardiovascular disease or cancer (30–34). Various metabolites are closely associated with multiple cellular processes, and disruptions in cell physiology caused by infectious or noninfectious diseases, which often alter metabolic footprints (35). Some studies report efforts to use metabolomic methods for disease diagnosis in pediatric patients (36–38). Metabolomics by evaluating a range of compounds in biological samples, offer potential for diagnosis, early disease detection, treatment selection as well as monitoring of therapeutic progress, and is promising in perinatal studies, such as hypoxic–ischemic encephalopathy (HIE), intrauterine growth restriction (IUGR), congenital infections, genetic diseases or neonatal nutrition (39, 40). The previous study by Danilewicz et al., showed the usage of untargeted metabolomics to compare blood metabolite profiles between pediatric patients with Chron’s disease (CD) or ulcerative colitis (UC) and healthy individuals for improving the noninvasive diagnostic (37). The study by Mickiewicz et al., with usage of nuclear magnetic resonance spectroscopy–based metabolomics distinguished several metabolites for recognition of early septic shock in children under the care of the pediatric intensive care unit. For instance, three compounds (2-hydroxybutyrate, 2-hydroxyisovalerate, and lactate) showed elevated levels in children with septic shock compared to healthy children regardless of differences in the age (36). Another study showed nine metabolites with diagnostic value for differentiation between children with asthma and healthy controls: adenine, adenosine, benzoic acid, hypoxanthine, p-cresol, taurocholate, threonine, tyrosine, and 1-methyl nicotinamide, which are associated with asthma related inflammatory processes. This metabolomic analysis also contributed to characterizing new asthma endotypes highlighting the heterogeneity of pediatric asthma (38). Cited examples of metabolomic studies on pediatric patients show variety of biomarkers differentiating diseases in children potentially specifically.

Our current research focuses on identifying systemic signatures of *H. pylori* infection in children by comparing sera from infected individuals with those from uninfected subjects. It may help explore metabolic changes associated with *H. pylori* infection in children and its potential impact on current and delayed systemic effects including growth retardation. We used ultra-performance liquid chromatography and quadrupole time-of-flight mass spectrometry (UPLC-QTOF/MS), a powerful platform for untargeted metabolomics. We expect that metabolomic analysis performed in this study will consist of introduction to further studies on diagnostic of *H. pylori* infection in children facilitating the identification of biomarkers for earlier detection of the disease, monitoring infection progression and personalized care, including treatment monitoring. However, standards and data-sharing initiatives are necessary as indicated by Sahu et al. (41).

## 2 Materials and methods

### 2.1 Patients and controls

Approval for this study was granted by the Bioethical Committee at the Polish Mother’s Memorial Hospital—Research Institute (PMMH-RI) in Lodz (RNN/134/13/KE/2-13). Baseline demographic and clinical characteristics of study groups is shown in **Table 1**. In total 64 children from Poland were involved into this study. Biological samples (serum) were obtained from: 32 children (both sexes -18 girls and 14 boys, mean age 12.5 ± 3.3) with gastrointestinal symptoms infected with *H. pylori* before specific medication – Hp (+). Children of this group were under the care of Department of Gastroenterology, Allergology and Pediatrics/Department of Endocrinology and Metabolic Diseases in PMMH-RI. Children with thyroid dysfunction, autoimmune diseases, eating disorders, chronic cardiovascular, respiratory, or urinary system diseases, as well as girls with Turner’s syndrome (diagnosed through chromatin X or karyotype tests), were excluded from the study. The inclusion criterion was infection with *H. pylori* confirmed by the presence of anti *H. pylori* IgG antibodies in serum samples) and gastroscopy-based reference diagnostic tests (rapid urease test, detection of Helicobacter-like organisms in gastric tissue thin layer preparations in conjunction with infiltration of inflammatory cells). Control group consisted of 32 healthy children (both sexes – 15 girls and 17 boys, mean age 11.5 ± 2.8) under the care of general medical care unit in PMMH-RI. The control group was selected based on the exclusion of gastrointestinal symptoms and negative result of non-invasive diagnostic tests for *H. pylori* (serological examination of serum samples for anti-*H. pylori* IgG or ^13^C UBT). None of the children from control group reported gastrointestinal symptoms or had a prior diagnosis or treatment for gastrointestinal diseases, including *H. pylori* infection.

**Table 1:** Baseline demographics and clinical characteristics of study groups.

Parameter/group	*H. pylori* positive group (Hp+)	*H. pylori* negative group – (Hp−)
Total number of participants	32	32
Sex (girls/boys)	18/14	15/17
Chronological age (years)	12.5 ± 3.3	11.5 ± 2.8
Clinical status	Gastrointestinal symptoms	Healthy, no gastrointestinal symptoms
Surgery intervention	No	No
Medication		
• recent antibiotics • specific medications • specific diet	No No No	No No No
*H. pylori* status	Positive	Negative
Inclusion criteria	Confirmed *H. pylori* infection	No gastrointestinal symptoms; no history of gastrointestinal diseases or *H. pylori* infection
Biological material collected	Serum	Serum

No recent uptake of antibiotics.

No specific medication usage before collecting serum.

Informed consent was obtained before the study, and participants’ privacy was protected. Blood samples were collected after fasting, upon admission, and before any medical or pharmacological treatment. Serum was separated within 1 hour, with 30 minutes of incubation at room temperature, followed by 30 minutes at 4 °C, then centrifuged at 2000× g for 10 minutes at 4 °C. The serum was aliquoted and stored at -80 °C for later analysis. Samples were thawed immediately before use in experiments.

### 2.2 Diagnosis of *H. pylori* infection in study groups

The establishment of *H. pylori* status in each group is shown in **Table 2**. The *H. pylori* status in patients with gastrointestinal symptoms was determined based on the ^13^C UBT (42) (some patients) or the laboratory enzyme-linked immunosorbent assay (ELISA) for detection of IgG antibodies against the glycine extract (GE) - antigenic complex containing surface antigens of the reference *H. pylori* strain CCUG (Culture Collection University of Gothenburg, Sweden, 17874), positive for CagA and VacA, as previously described (43). The GE protein concentration was 600 μg/mL (NanoDrop 2000c Spectrophotometer, Thermo Scientific, Waltham, MA, USA), while lipopolysaccharide (LPS) concentration was below 0.001 EU/mL, as shown by the chromogenic Limulus amebocyte lysate test (Lonza, Braine-l’Alleud, Belgium).

**Table 2:** Major diagnostic tests for *H. pylori* infection.

Parameter/Group	*H. pylori* positive group (Hp+)	*H. pylori* negative group - control (Hp−)
^13^C UBT	Positive	Negative
Serological test: - ELISA for anti-*H. pylori* IgG antibodies Coating antigen:		
• GE	Positive OD 450>0.3	Negative OD 450<0.3
• CagA	Positive 14/32 OD 450>0.3	Not tested
-Western blot for anti *H. pylori* IgG antibodies	Positive	Not tested
Gastroscopy based diagnostic tests: - RUT - HLO (Giemsa staining) - Inflammation (H&E staining)	Positive	Not tested Not tested Not tested

ELISA, Enzyme-Linked Immunosorbent Assay; ^13^C UBT, Urea Breath Test; HLO, Helicobacter-like organism; H&E, Hematoxylin and Eosin; GE, glycine extract; CagA, Cytotoxin-Associated Gene A; VacA, Vacuolating Cytotoxin A; Hsp, Heat shock protein; Ure, urease subunit; RUT, Rapid Urease Test.

Serum samples were also tested for IgG antibodies against *H. pylori* cytotoxin associated gene A (CagA) protein using recombinant CagA protein (rCagA) (courtesy of Dr Antonello Covacci, IRIS, Siena, Italy), as previously described (44). The ELISA test for anti-*H. pylori* IgG antibodies was validated using the pools of reference serum samples from children with *H. pylori* infection confirmed or excluded by gold standard gastroscopy-based tests: rapid urease test, histological examination for detection HLO and assessment of inflammatory response, as well as microbial culture. The *cut off* absorbance value (OD 450 nm) in the ELISA for anti-*H. pylori* GE IgG or anti *H. pylori* CagA IgG was >0.3.

The ELISA results were verified concerning specificity of IgG antibodies to *H. pylori* antigens by immunoblotting (Milenia^®^Blot *H. pylori*, DPC Biermann, GmbH, Bad Nauheim, Germany). Major proteins in GE, which were recognized by the serum antibodies from *H. pylori*-infected individuals included: CagA - 120 kDa, VacA - 87 kDa, urease subunit B (UreB) - 66 kDa, heat shock protein (Hsp) - 60 kDa, urease subunit A (UreA) - 29 kDa, and proteins between 66–22 kDa (44). Additionally, children with dyspeptic symptoms, underwent gastroscopy and routine histological examination of gastric tissue samples for HLO and infiltration of inflammatory cells. Gastric tissue specimens were also used for development of rapid urease test (Lencomm Trade International, Warsaw, Poland). *H. pylori* exposure in children without gastrointestinal symptoms - Hp (–) was excluded based negative result of ELISA for anti-*H. pylori* IgG antibodies or ^13^C UBT (some individuals). The coincidence between the ELISA for anti-*H. pylori* IgG and ^13^C UBT and between ELISA and Immunoblot was 98% as previously described (44).

### 2.3 Metabolomic analysis

#### 2.3.1 Materials

LC–MS grade solvents: acetonitrile (ACN), methanol (MeOH) (J.T. Baker, Avantor Performance Materials, Gliwice, Poland) and LC–MS-grade mobile phase modifiers: formic acid (FA) (Chem-LAB NV, Zedelgem, Belgium) was applied. Ultra-high-purity water was prepared using the R5 UV Hydrolab system (Wislina, Poland). Internal standard (IS) mixture reagents were benzoyl-D5 (98%) and L-phenylalanine 3,3-D2 (98%) (Cambridge Isotope Laboratories, Inc., Tewksbury, MA, USA).

ELISA - Enzyme-Linked Immunosorbent Assay; ^13^C UBT - Urea Breath Test, HLO – Helicobacter-like organism, H&E - Hematoxylin and Eosin, GE – glycine extract, CagA - Cytotoxin-Associated Gene A, VacA - Vacuolating Cytotoxin A, Hsp - Heat shock protein, Ure, RUT- Rapid Urease Test.

#### 2.3.2 Metabolomic procedures

##### 2.3.1.1 Sample collection and processing

After thawing at 4 °C, 100 μL of serum was transferred and mixed with 300 μL of ice-cold acetonitrile solution containing IS. The diluted serum samples were incubated for 20 min at -20 °C and then centrifuged (20,000 × g, 10 min, 4 °C). Next, 200 µL of supernatant was aliquoted into low-recovery-volume HPLC vials. Quality control (QC) samples were prepared by mixing equal aliquots of all thawed and centrifuged serum samples. The analytical batch included 10 samples for system equilibration, 9 QC samples, 62 serum samples (32 from *H. pylori*-infected and 32 from non-infected children), and 2 blank samples. Quality control (QC) samples were prepared by combining equal volumes of aliquots from every serum sample and used to monitor system stability (injected every ten analyzed samples). More detailed information on sample analysis is available in our previous study (45).

##### 2.3.1.2 Instrumental analysis - LC/MS analysis

For the assessment of serum metabolomic profile, an untargeted metabolomic analysis with the analytical system consisting of Waters Acquity™ UPLC (Waters Corp., Milford, MA, USA) connected to a Synapt G2Si QTOF/MS spectrometer (Waters MS Technologies, Manchester, UK), equipped with an electrospray source (ESI), (Waters MS Technologies, Manchester, UK) was used. Metabolite separation was executed using ACQUITY UPLC BEH C18 precolumn (1.7 µm, VanGuard Precolumn 2.1 × 5 mm) connected with an ACQUITY UPLC BEH C18 (1.7 µm, 2.1 × 100 mm) chromatography column (Waters, Milford, MA, USA) for positive and negative ionization modes. The mobile phases were as follows: (A) 0.1% FA in water and (B) 0.1% FA in ACN. The injection volume was 3 µL for positive mode and 6 µL for negative mode; the temperature was maintained at 40 °C, and the flow was 2.5 mL/min (both in positive and negative modes). **Supplementary Table 1** shows the optimized gradient elution procedures.

All analyses were performed in MS centroid, high-resolution mode with a time scan of 0.3 s. The gas flows were 900 L/h for the desolvation gas, 100 L/h for the cone gas, and 6.5 Bar for the nebulizer. Temperatures were 350 °C for desolvation and 120 °C for the source. The capillary voltage was 3.2 kV (positive mode) and 2.4 kV (negative mode). To ensure accuracy and reproducibility, the lock mass (leucine-enkephalin) was used with the following settings: scan time 0.5 s., interval 15 s., scans to average: 3, and mass window ± 0.5 Da.

##### 2.3.1.3 Data analysis (metabolomic data preprocessing, normalization)

Progenesis QI v3.0 software (Waters, Milford, MA, USA) was used for processing untargeted LC/MS data files. The raw data included 7159 features in positive mode and 5869 in negative mode. Feature detection, retention time correction, alignment, and putative annotation of compound classes were analyzed using the default parameter settings for UPLC – High Resolution (Waters), achieving level 3 annotation (n = 1996 in positive mode, n = 1271 in negative mode) according to the minimum reporting standards defined by the Metabolomics Standards Initiative (46). Data with level 3 annotation acquired from Progenesis QI v3.0 software were filtered. Metabolite features were discarded based on such criteria: a blank contribution greater than 5%, missing values exceeding 50%, and a QC relative standard deviation (RSD) above 25%. After cleaning, the final biomarker selection included 597 features in positive mode and 640 features in negative mode. Signal drift correction, and normalization were performed on the remaining data using the MetaboGroupS software application (https://www.omicsolution.com/wukong/MetaboGroupS/) (46). Missing values in the data were imputed with the k-nearest Neighbor (KNN) algorithm within MetaboGroupS software, followed by log2 transformation to correct data skewness. common normalization methods (median normalization, standard normalization, variance-stabilizing normalization, removal of unwanted variation-random normalization, QC sample-based support vector regression, EigenMS, and QC sample-based support vector regression) were evaluated. We chose the EigenMS method with the lowest entropy coefficient of variation (CV) in QC samples to normalize both positive and negative modes (**Supplementary Figures 1**, **2** in the **Supplementary File**) (47).

##### 2.3.1.4 Metabolite annotation

To annotate statistically significant compounds, a fragmentation procedure was used. The same LC/MS conditions were applied to both ESI + and ESI − modes, and, in addition, a collision energy ramp from 20 to 60 V was applied (FASTDDA method). Received data files (.row) were converted to mzML using MSConvert v. 3.0.25002-23ff91c. For annotation, the received mzML data files were loaded into the MSDIAL console v5.5.241113. Parameters of MS-DIAL for annotation of compounds are in the **Supplementary Materials** (**Supplementary Tables 2**, **3**). The fragmentation spectra were matched against those available in the Human Metabolome Database (48) for level 2 putative annotation of compounds. Parameters of MS-DIAL for annotation of compounds are in the **Supplementary Materials** (**Supplementary Tables 2**, **3**).

### 2.4 Statistical analysis

Normalized data were subjected to statistical analysis to identify metabolites that differentiated *H. pylori*-infected from non-infected children. These differences in the signal intensity were analyzed using the unpaired t-test (p-value threshold = 0.05) as well as Fold Change (FC) analysis (FC threshold = 1.15). These results were combined into two graphs (in positive and negative ionization mode) as a volcano plot method with the MetaboAnalyst online platform (https://www.metaboanalyst.ca/). The results were compared to find potential metabolites that differentiated the examined groups. In the case of selected putatively annotated metabolites, whose signal intensity was significantly increased in serum samples from *H. pylori*-infected children vs. serum samples from uninfected individuals, additional information about the adjusted p-value was added. Classical univariate receiver operating characteristic (ROC) curve analyses were used to identify potential biomarkers for determining the analyzed groups. Classical ROC analysis is frequently applied to algorithms for building ROC curves and calculating the area under the curve (AUC) to compute optimal metabolite cut-offs and generate sensitivity and specificity data. These classical univariate ROC curve analyses were generated in the MetaboAnalyst 5.0 platform (https://www.metaboanalyst.ca) and accessed on 23 October 2025.

## 3 Results

### 3.1 *H. pylori* status

**Figure 1:**
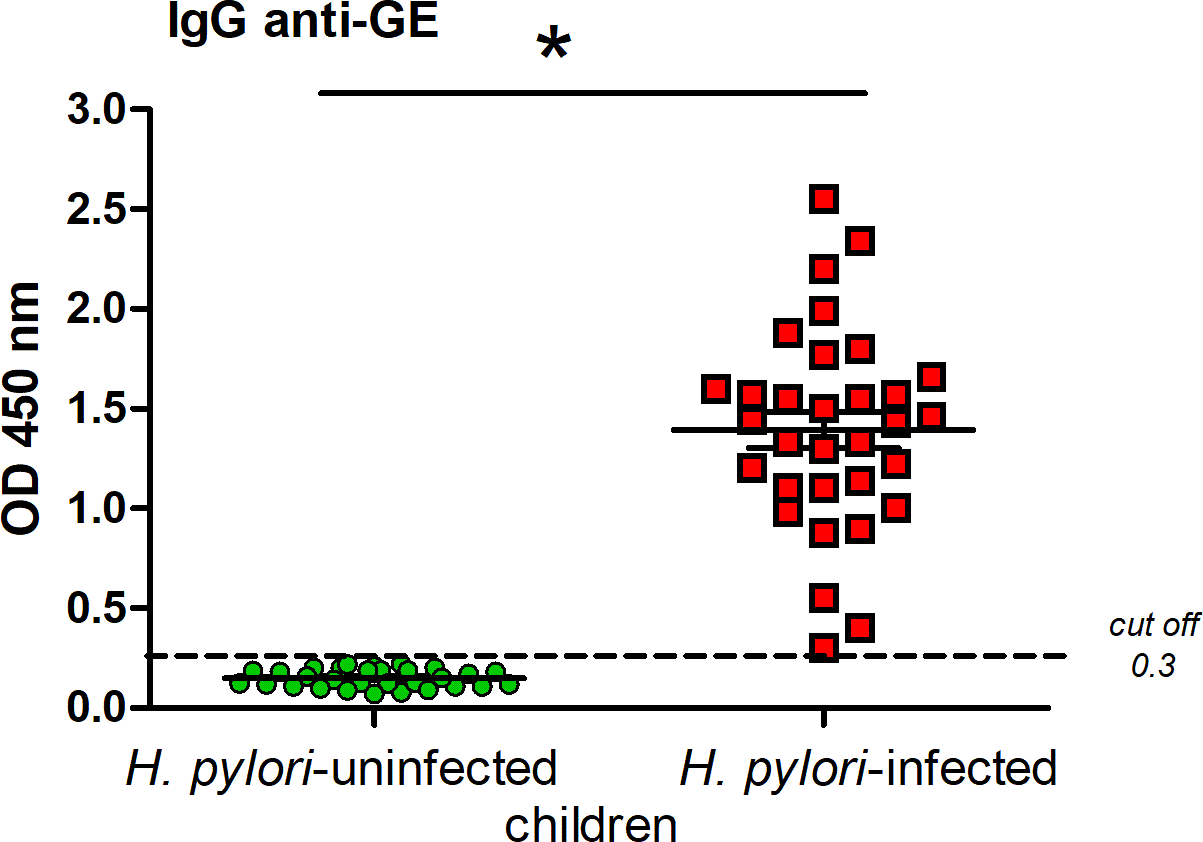
The prevalence and levels of anti*-H. pylori* IgG antibodies in children under the study. The level of anti-*H. pylori* antibodies was assessed by enzyme-linked immunosorbent assay (ELISA) using antigenic complex - glycine acid extract of the reference *H. pylori* strain. Children seronegative for anti-*H. pylori* antibodies—*H. pylori* uninfected, n = 32; Children infected with *H. pylori* as confirmed by gastroscopy-based testing - seropositive for anti-*H. pylori* antibodies—*H. pylori* infected, n = 32. Shown are mean values + SEM. *Statistical significance p>0.05, *H. pylori* uninfected vs. *H. pylori* infected.

To distinguish between groups, serum samples from healthy children without dyspeptic symptoms non infected with *H. pylori* – Hp (–) and individuals with gastrointestinal symptoms *H. pylori*-infected – Hp(+) were analyzed using ultra-performance liquid chromatography coupled with quadrupole time-of-flight mass spectrometry (UPLC-QTOF/MS). This approach aimed to search metabolomic biomarkers that can differentiate serum samples from *H. pylori* infected from *H. pylori* non-infected children and detect products resulting from *H. pylori*-induced cellular activities. Serum panels included samples from healthy children—32 seronegative for anti-*H. pylori* IgG and from 32 children with *H. pylori*-induced gastritis, confirmed by histological examination in conjunction with the presence of HLO and seropositive for anti-*H. pylori* IgG. These samples were used for metabolomic analysis (**Figure 1**).

### 3.2 Metabolomic analysis

**Figure 2:**
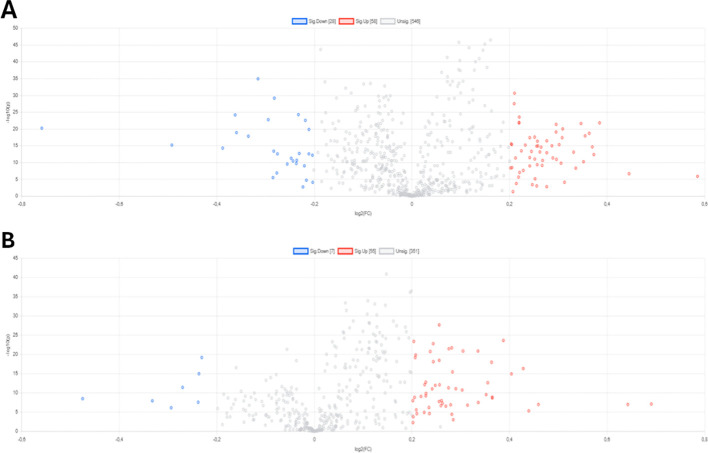
The volcano plot shows changes in signal intensity for metabolites between *H. pylori*-infected **(A)** vs. *H. pylori* uninfected children **(B)**. Metabolites with significantly higher signal: red; metabolites with significantly lower signal: blue.

The exploratory metabolomic analysis of children’s serum samples performed in this study allowed selection of several biomarkers potentially associated with *H. pylori* infection. We found15 metabolites with significantly decreased signal intensities and 43 metabolites with notably increased signals in *H. pylori*-infected children compared to uninfected ones, as illustrated by Volcano plots (**Figure 2**). Among the metabolites which allowed differentiating the serum samples of children infected with *H. pylori* from children uninfected with these bacteria we found: carboxyethyl lysine - CEL (HMDB29447), gamma-Glutamylleucine – (Gamma-Glu-Leu) (HMDB0011171), 13-HOTrE(y) hydroxylated and oxidized derivative of the omega-6 fatty acid arachidonic acid (HMDB0341541), 13-HODE (HMDB0004667), lauroylcarnitine (HMDB0002250), vitamin A (HMDB0000305) and 19_norandrosterone (HMDB0002697). In **Table 3**, 7 metabolites with significantly higher signals, associated with *H. pylori* infection, are presented.

**Table 3:** Selected putatively annotated metabolites whose signal intensity was significantly increased in serum samples from *H. pylori-*infected children vs. serum samples from uninfected individuals.

Lp	log2(FC)	FC	*m*/*z*	RT (min)	*p*-value (adjusted *p*-value)	Name (ID in HMDB)	Locations	Biological process
1	0.25262	1.1914	217.1191	1.17	7.04E-06 (1.06E-05)	carboxyethyl lysine (HMDB29447)	Extracellular Cytoplasm	contribute to inflammation and oxidative stress promotes lipid uptake by macrophages (44)
2	0.30581	1.2361	259.1296	2.12	1.61E-10 (3.17E-10)	gamma-Glutamylleucine (HMB0011171)	Non-excretory biofluid	associated with inflammation, oxidative stress, and glucose regulation, and is causally linked to increased risks of cardio-metabolic diseases like obesity and type 2 diabetes (45)
3	0.28698	1.2201	293.2117	4.18	1.03E-15 (3.05E-15)	13-HOTrE(y) (HMDB034154)	No data	Increases the expression of the anti-inflammatory cytokine IL-10 reduces the expression of pro-inflammatory cytokines, including IL-1β and IL-6 (46)
4	0.36377	1.2868	295.2274	4.07	1.87E-19 (7.91E-19)	13-HODE (HMDB000466)	Membrane Extracellular	prevents cell adhesion to endothelial cells and can inhibit cancer metastasis. Involved in cell proliferation and differentiation (47)
5	0.22012	1.1648	342.2646	7.41	2.85E-24 (2.08E-23)	lauroylcarnitine (HMDB0002250)	Membrane Extracellular Cytoplasm Mitochondrion matrix Cytoplasm Cell membrane	production of energy by transporting fatty acids into mitochondria (48)
7	0.22887	1.1719	329.2475	5.87	1.39E-10 (3.33E-10)	vitamin A acetate (HMDB000030)	Cytoplasm Extracellular Membrane (predicted from logP)	forming rhodopsin helps regulate gene expression, cell proliferation, differentiation, and maintains epithelial tissues throughout the body (49)
8	0.40398	1.3232	277.2162	6.17	1.21E-15 (4.36E-15)	h_14_19_norandrosterone (HMDB000269)	Membrane Extracellular Cytoplasm Cell membrane	

**Figure 3:**
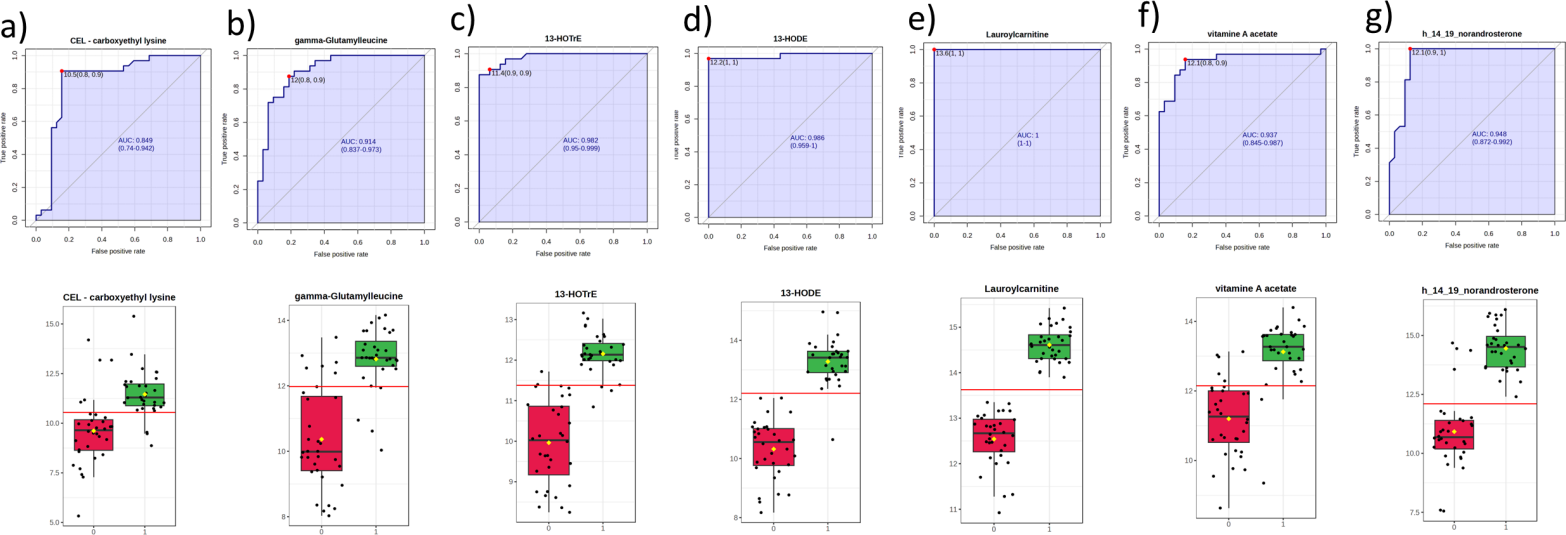
Classical univariate receiver operating characteristic curves generated from the spectral data to identify serum metabolomic biomarkers related to *H. pylori* infection. Box plots representing the distribution of the normalized signal intensities come from: **(A)** carboxyethyl lysine (HMDB29447); **(B)** gamma-Glutamylleucine (HMDB0011171); **(C)** 13-HOTrE (y) – hydroxylated and oxidized derivative of the omega-6 fatty acid arachidonic acid (HMDB0341541); **(D)** 13-HODE (HMDB0004667); **(E)** lauroylcarnitine (HMDB0002250); **(F)** vitamin A (HMDB0000305); and **(G)** 19-norandrosterone (HMDB0002697). The boxes represent the interquartile range (difference between the upper 75% and the lower 25%), the thick black lines represent the median and the thin black lines represent the upper and lower quartiles. A horizontal red line indicates the optimal cutoff. Abbreviations: 0—*H. pylori-negative*; 1 – *H. pylori* positive.

The classical univariate ROC curve analysis performed in this study showed that the selected biomarkers facilitated preliminary distinguishing children infected with *H. pylori* from those not infected (**Figure 3**). We found that potential serum biomarkers for *H. pylori*-infected individuals had an Area Under the Curve (AUC) exceeding 0.9, indicating a statistically significant difference between the two groups (*H. pylori* infected vs. *H. pylori* non-infected) (**Figure 3**, **Supplementary Table 3**).

## 4 Discussion

*H. pylori* cause gastritis, gastric or duodenal ulcers, and consists of the risk for the development of gastric cancer. The reasons behind these varying responses to *H. pylori* remain unclear. In children, chronic infection by impacting nutritional balance may correlate with malnutrition and growth delay (21, 50–53). In *H. pylori* infected children, deficiencies in macro- and micronutrients, including iron, zinc, selenium, vitamin C, vitamin A, tocopherol, vitamin B12, and folic acid, as well as essential minerals are quite common (54–60). Therefore, it has been recommended to monitor the levels of these biomarkers in relation to *H. pylori* infection.

In our previous study based on IR spectra analyses of serum samples from *H. pylori*-infected vs. uninfected children, we selected 10 wavenumbers correlating with *H. pylori* infection, which were compatible with vitamin A, vitamin B6, vitamin B12, vitamin C, tocopherol, folic acid, carotene, and lutein, as well as the hormone peptides ghrelin and leptin. These wavenumbers were selected for Artificial Neural Network (ANN) design for rapid diagnosis of *H. pylori* infection in children (61). These results and those of other authors indicate the potential of combining various physicochemical methods, e.g. FTIR and metabolomic methods in modern diagnostics facilitating biomarker discovery or focusing on specific metabolites (62). The study by Daniluk et al., revealed that inflammatory diseases such as CD and UC have an impact on lipid metabolism in pediatric patients. Lactosylceramide (LacCer 18:1/16:0) has been shown as a unique metabolite significantly increasing in CD patients’ sera which, along with inflammatory markers including C-reactive protein (CRP) can discriminate children with CD from UC with high specificity and sensitivity (37).

In this exploratory study, we employed ultra-performance liquid chromatography combined with quadrupole time-of-flight mass spectrometry (UPLC-QTOF/MS) to search metabolomic biomarkers that may monitor *H. pylori* infection in children and detect products of *H. pylori*-induced cellular activities. Serum samples from 32 individuals, without gastrointestinal symptoms, non-infected with *H. pylori*, seronegative for anti-*H. pylori* IgG and 32 children infected with *H. pylori* with *H. pylori*-induced gastritis seropositive for anti-*H. pylori* IgG were analyzed. We identified 15 metabolites with significantly lower and 43 with higher signal intensities in infected children, as shown on Volcano plots. Metabolites with differentiating potential include carboxyethyl lysine (CEL), gamma-Glutamylleucine, 13-HOTrE, 13-HODE, lauroylcarnitine, vitamin A, and 19_norandrosterone. Database literature searching indicated that these 7 selected metabolites, which potentially can differentiate children’s sera from *H. pylori* infected vs. uninfected individuals, are associated with immune regulation, energy metabolism, lipid/fatty acid metabolism, lipid peroxidation, oxidative stress, and cell signaling, and correlate with the pathogenesis of *H. pylori* infection in humans. The remaining 36 metabolites may be considered additional markers of *H. pylori* infection; however, their role during *H. pylori* pathogenesis has not been described.

Carboxyethyl lysine (CEL) selected by us as potential biomarker differentiating *H. pylori* positive vs. *H. pylori* negative group is a lysine derivative with potential clinical relevance. High levels of CEL are associated with various diseases including diabetes, cardiovascular diseases and symptoms of chronic inflammation (63–65), the state which was confirmed in *H. pylori* infected children during histopathological examination of gastric tissue specimens. The buildup of CEL caused by hyperglycemia and enhanced glycosylation can induce inflammation and oxidative stress, potentially accelerating disease progression. Studies on CEL’s effects on cellular DNA suggest that it could harm the integrity of the genome (66). Cellular experiments confirm that CEL can cause DNA damage and chromosomal abnormalities (67), thereby raising the risk of genotoxicity and mutation. This finding is important concerning the risk of gastric cancer in *H. pylori* infected individuals. Early *H. pylori* infection in childhood, which then lasts for decades by resulting in CEL overexpression, may potentially link this infection with development of pro-cancerous environment. In this context CEL sims to be an important metabolomic biomarker in *H. pylori* infected children.

13-HOTrE- hydroxylated and oxidized derivative of the omega-6 fatty acid arachidonic acid, 13-HODE are stable, abundant oxidation products detectable in human plasma. They have been associated with a range of pathological conditions, including inflammation dependent dysregulation related to atherogenesis, cancer, metabolic syndrome, and other disease processes (68–70). 13 HODE are involved in signaling and regulating inflammatory processes, including cell adhesion, neutrophil chemotaxis and degranulation, macrophage superoxide production, PPAR-γ activation, and inhibition of protein kinase C (70–73). *H. pylori* infection can induce the advanced glycation end-products (AGEs) such as CEL, although this area is less studied compared to other risk factors. CEL is derived from methylglyoxal reacting with lysine and is linked to oxidative stress markers like 13-HODE, which *H. pylori* infection induces in the stomach. The infection triggers an inflammatory response that delivers reactive oxygen species (ROS), leading to oxidative stress that can harm host cells and contribute to diseases such as gastric cancer (74–76). *H. pylori* LPS is a strong proinflammatory bacterial stimulus generating ROS resulting in disintegration and apoptosis of gastric epithelial cells *in vitro* and *in vivo* as shown in biological model of *H. pylori* infection in *Cavia porcellus*. The recovery of gastric barrier cells during *H. pylori* infection potentially can be affected due to downregulation of pro-regenerative activity of interleukin (IL-33) by *H. pylori* LPS (77).

Recent data indicate that gamma-glutamyl dipeptides play a role in various biological processes, such as inflammation, oxidative stress, and glucose regulation, through activating calcium-sensing receptors (CasR) in different organs (78). CasR influences numerous cellular functions related to cardiovascular health, including insulin secretion, nitric oxide release, apoptosis, cell proliferation, and NOD-, LRR- and pyrin domain-containing protein 3 (NLRP3) inflammasome activation. Epidemiological data also link abnormal gamma-glutamyl dipeptide levels to multiple conditions like obesity, metabolic syndrome, type 2 diabetes, non-alcoholic fatty liver disease, and cardiovascular diseases (84). Increased gamma-glutamyl leucine dipeptide potentially relates to *H. pylori* infection through bacterial gamma-glutamyl transpeptidase (GGT)processing glutamine and glutathione. GGT secreted by *H. pylori* may affect host cell growth, increase oxidative stress and inflammation, possibly leading to the development of gastric ulcer or gastric cancer indirectly, via the breakdown of gamma glutamyl leucineamino acids by GGT (79).

It has been found that lauroylcarnitine a derivative of L-carnitine, inhibits AMP-activated protein kinase (AMPK) activation, a pathway that promotes the anti-inflammatory M2 macrophage phenotype plays a role in the host’s response to infection (80, 81). During *H. pylori* infection the enhanced release of L-carnitine may represent the host response towards elevated oxidation to prevent gastric lining.

The results obtained in this study showed that lauroylcarnitine (HMDB0002250) may potentially be candidate as diagnostic marker due to AUC = 1. According to binary analysis (sick/healthy) this marker differentiates properly Hp(+) from Hp (–) individuals (100% specificity and sensitivity) despite of cut off. However, the small number of patients per group (n=32) consists of limitations of this finding. The higher number of patients in the future study will facilitate validation of this data using adequate mathematical model to avoid “overfitting”.

Nandrolone, or 19-nortestosterone, is a synthetic anabolic-androgenic steroid (AAS) belonging to steroids’ family and naturally derived from the testosterone molecule, the primary sex steroid hormone produced in men (82). Studies suggest that *H. pylori* can metabolize steroid hormones, which may be associated with lower overall androgen activity. *H. pylori* can absorb and utilize hormones such as epiandrosterone and dehydroepiandrosterone to synthesize cell membrane lipids. Still, it does not adsorb or utilize androsterone, the primary metabolite of nor androsterone (83).

*H. pylori* infection also can lead to deficiencies in several vitamins, including vitamin A, C, B12, and E, due to malabsorption, inflammation, and altered gastric pH (79). Furthermore, molecular mimicry between *H. pylori* and growth hormones regulating appetite and food intake, including ghrelin, leptin, orexin or alpha-melanocyte-stimulating hormone (alpha-MSH) may potentially lead to production of autoantibodies cross-reacting with growth hormones and their deficiency (84).

Although the reference diagnostic tests for confirming *H. pylori* infection are sensitive and specific, they do not facilitate monitoring systemic metabolic effects, which may be related to the course of infection. Metabolomic LC-MS analysis of serum samples from *H. pylori* infected vs. *H. pylori* uninfected children potentially may have clinical relevance. It could help select soluble systemic biomarkers related to gastric barrier dysfunction due to *H. pylori* driven inflammation and these biomarkers may potentially be related to the late systemic effects of infection, which, however, requires further research to confirm this hypothesis. A higher number of biological samples is necessary to standardize metabolomic techniques for diagnostic-medical application.

## 5 Conclusions

By using exploratory metabolomic analysis of serum samples combined with ROC analysis, we selected 7 signatures potentially associated with *H. pylori* infection.

The metabolites differentiating serum samples from *H. pylori* infected children vs. serum samples from *H. pylori* negative children include: carboxyethyl lysine (HMDB29447); gamma-Glutamylleucine (HMDB0011171); 13-HOTrE (y) – hydroxylated and oxidized derivative of the omega-6 fatty acid arachidonic acid (HMDB0341541); 13-HODE (HMDB0004667); lauroylcarnitine (HMDB0002250); vitamin A (HMDB0000305); and 19-norandrosterone (HMDB0002697). Based on databases and literature these metabolites are related to progression or regulation of inflammatory response, cell signaling, cell proliferation and differentiation, maintaining epithelial barriers, lipid metabolism or DNA degradation. It is consistent with processes induced by *H. pylori* including inflammatory response being deleterious to gastric barrier, modulation the activity of immunocompetent cells, influence to lipid metabolism, induction of oxidative stress and DNA degradation. It is not known whether there is a link between selected metabolites and different clinical outcomes of *H. pylori* infection in children. This exploratory study delivered preliminary results on serum metabolomic profiling combined with ROC analysis which potentially may facilitate differentiation *H. pylori*-infected from uninfected children. Whether selected metabolites indicate the systemic effects of chronic infection remains unclear. Identified metabolites are linked to many aspects of immunity and inflammation or even broader systemic consequences of chronic *H. pylori* infection, however the study is relatively small, and does not directly measure immune phenotypes, inflammatory mediators, growth outcomes, or longitudinal clinical endpoints therefore these connections consist of interesting hypotheses, which should be verified in further study, including validation of proposed methodology. Additional validation (e.g., targeted LC-MS analysis or correlation with clinical parameters) would be of great importance.

Future studies should explore whether metabolomic parameters identified here are reliable biomarkers reflecting *H. pylori* driven local and systemic effects. If yes, including these additional parameters in diagnostic analyses would potentially help to better understand the endogenous mechanisms of *H. pylori* infection and enable a more thorough assessment of patient health status, and may help develop targeted treatment options.

It is worth mentioning that 7 metabolites selected in this preliminary study may then be used as internal markers in FTIR method in conjunction with mathematical ANN model for ANN learning to differentiate *H. pylori* infected from *H. pylori* uninfected children based on monitoring IR serum spectra.

## Data Availability

The datasets presented in this study can be found in online repositories. The names of the repository/repositories and accession number(s) can be found in the article/**Supplementary Material**.
