# Supplementary material for: Exploratory metabolomic analysis for identifying systemic signatures of Helicobacter pylori infection in children
Source: Front Immunol. 2026 Jun 16;17:1826604. doi: 10.3389/fimmu.2026.1826604 (PMC13314522; doi:10.3389/fimmu.2026.1826604)
Supplement: Supplementary file 1 [file DataSheet1.docx]

Supplementary Material

**Exploratory metabolomic analysis for identifying systemic signatures of *Helicobacter pylori* infection in children**

Weronika Gonciarz, Lucyna Kozlowska, Marta Stelmasiak, Magdalena Wozniczka Magdalena Chmiela

**Supplementary Table S1.** The optimized gradient elution procedure (the same for positive and negative mode)

| Time | Flow (mL/min) | %A1 | %B1 | Curve |
| --- | --- | --- | --- | --- |
| Initial | 0.25 | 99 | 1 | Initial |
| 1.00 | 0.25 | 75 | 25 | 6 |
| 2.00 | 0.25 | 55 | 45 | 6 |
| 3.00 | 0.25 | 40 | 60 | 6 |
| 4.00 | 0.25 | 26 | 74 | 6 |
| 10.00 | 0.25 | 20 | 80 | 6 |
| 11.00 | 0.25 | 0 | 100 | 6 |
| 12.00 | 0.25 | 99 | 1 | 6 |
| 15.00 | 0.25 | 99 | 1 | 6 |

**Supplementary Table S2** Data processing parameters of MS-DIAL software used in this study.

| **Data collection parameters:** |
| --- |
| Mass accuracy: MS1 tolerance for centroid: 0.01 Da  MS2 tolerance for centroid: 0.025 Da |
| Retention time begin: 0 min  Retention time end: 100 min |
| MS1 Mass range begin: 50 Da  MS1 Mass range end: 1200 Da |
| MS/MS Mass range begin: 30 Da  MS/MS Mass range end: 1200 Da |
| **Peak detection parameters:** |
| Minimum peak height: 100 amplitude  Minimum slice width: 0.05 Da |
| **Smoothing method:** Linear weighted moving average  Smoothing level: 3 scan  Mass slice width: 5 scan |
| **Spectrum deconvolution:** |
| Sigma window value: 0.5  MS/MS abundance cut of: 0 amplitude  Exclude after precursor – yes  Keep the isotopic ions unit: 5 Da |
| **Identification:** |
| Accurate mass tolerance (MS1) for peak identification: 0.01 Da  Accurate mass tolerance (MS2) for peak identification: 0.025 Da |
| **MS2 spectrum cut off:** |
| Spectrum amplitude cutoff (relative): 0%  Spectrum amplitude cutoff (absolute): 0  Mass range begins: 0 Da  Mass range end: 1200 Da |
| **Alignment parameters setting:** |
| Retention time tolerance: 0.1 min  MS1 tolerance: 0.01 Da  Retention time factor: 0.5  MS1 factor: 0.7 |

Adduct ion setting:

| Negative mode | Positive mode |
| --- | --- |
| [M-H]^-^ | [M+H]^+^ |
| [M-H2O-H] | [M+NH4]^+^ |
| [M+Na-2H]^-^ | [M+Na]^+^ |
| [M+Cl]^-^ | [M+CH3OH+H]^+^ |
| [M+K-2H]^-^ | [M+K]^+^ |
| [M+HCOO]^-^ | [M+ACN+H]^+^ |
| [M+CH3COO]^-^ | [M+H-H2O]^+^ |
| [2M-H]^-^ | [M+H-2H2O]^+^ |
|  | [M+2Na-H]^+^ |
|  | [M+ACN+Na]^+^ |
|  | [M+2K-H]^+^ |
|  | [2M+H]^+^ |
|  | [2M+NH4]^+^ |
|  | [2M+Na]^+^ |
|  | [2M+K]^+^ |
|  | [2M+ACN+H]^+^ |
|  | [2M+ACN+Na]^+^ |


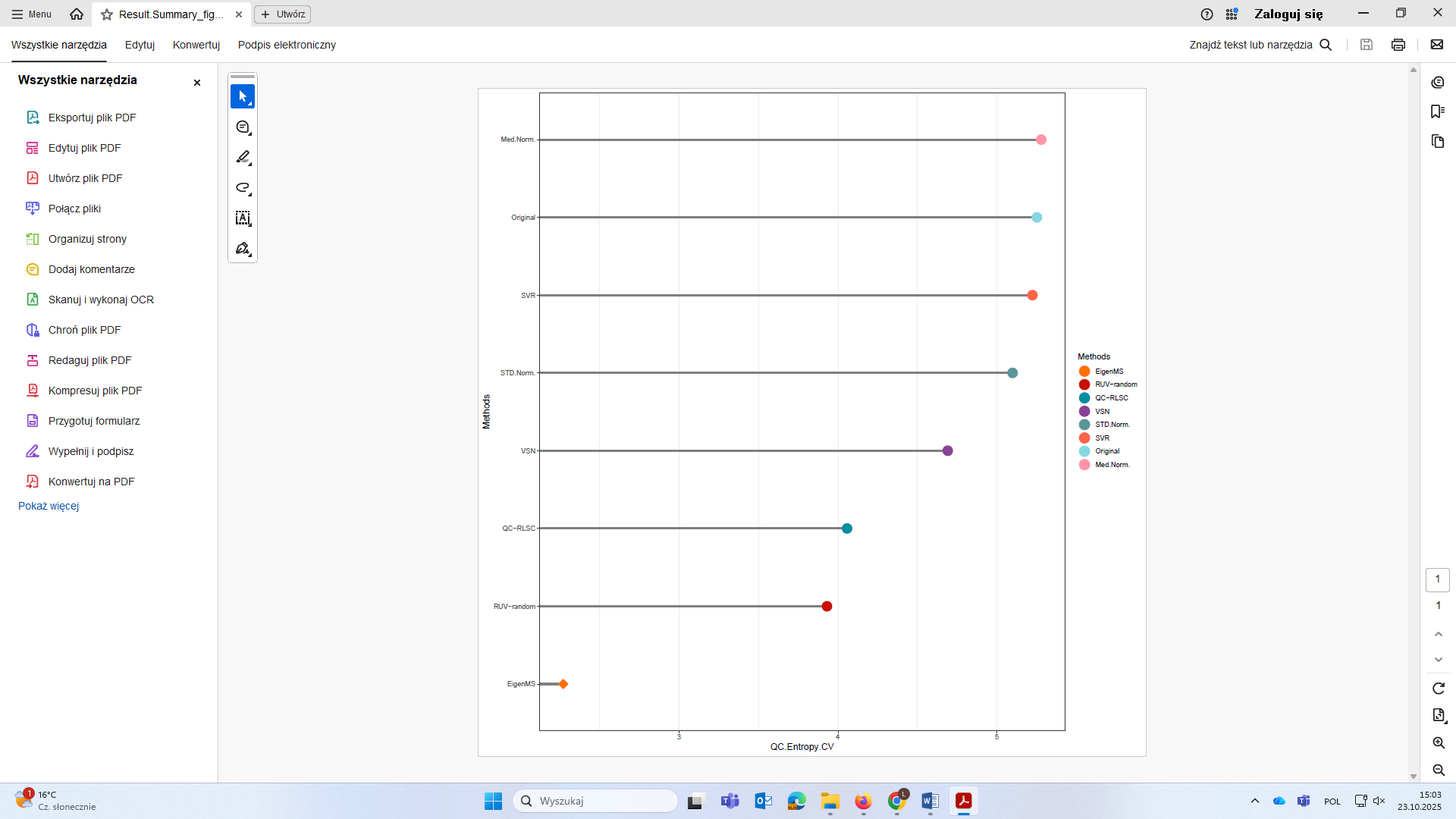


**Supplementary Figure S1.** Summary results of coefficients of variation (CV) of entropy in QC samples with respect to no normalization and seven normalization methods in negative MS mode.

Abbreviations: EigenMS - singular value decomposition-based normalization; QC-RLSC - QC sample-based support vector regression; VSN - variance-stabilizing normalization; MedNorm - median normalization; STD.Norm. - standard normalization; Original – no normalization; RUV-random - removal of unwanted variation-random normalization


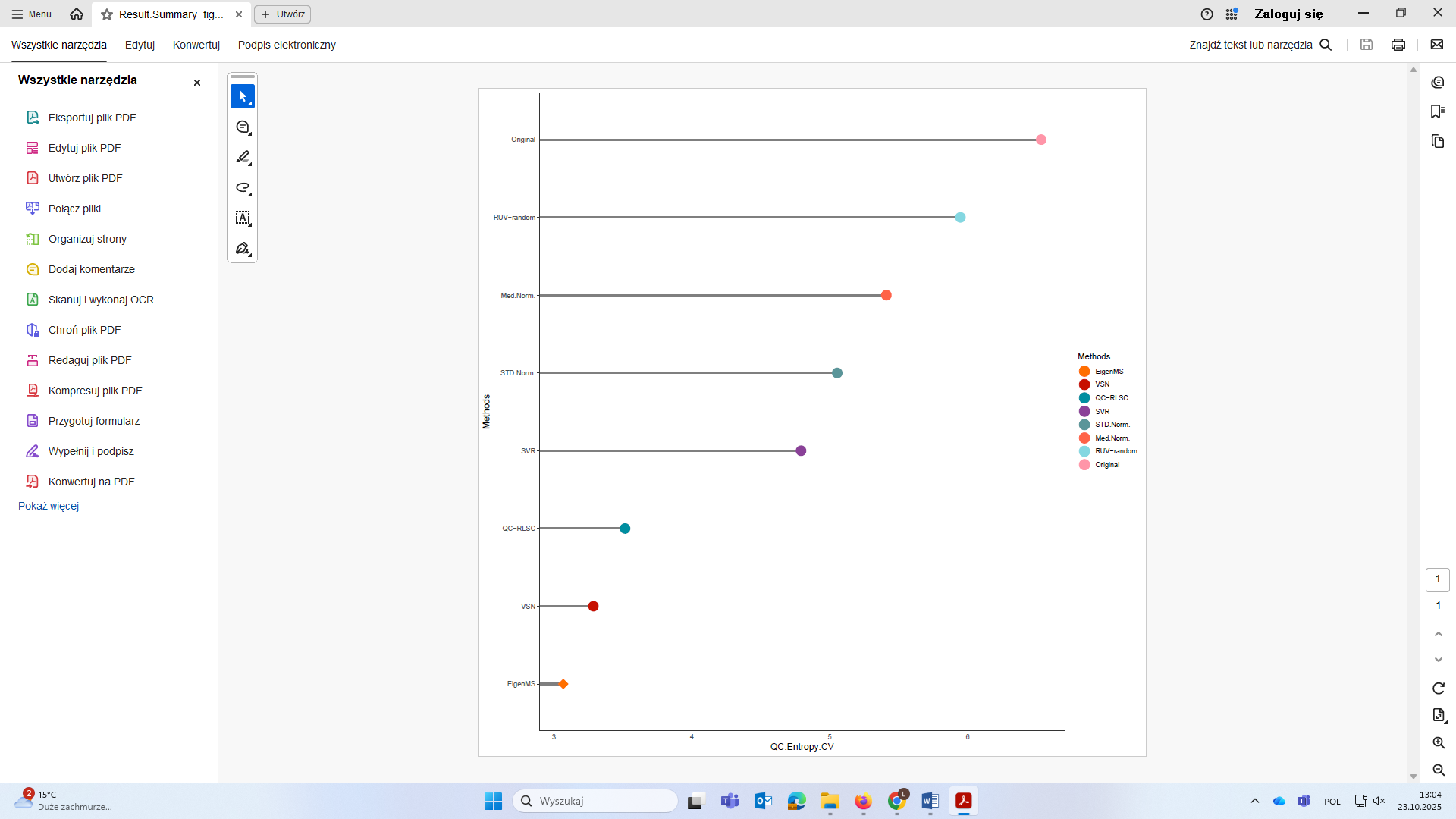


**Supplementary Figure S2.** Summary results of coefficients of variation (CV) of entropy in QC samples with respect to no normalization and seven normalization methods in positive MS mode.

Abbreviations: EigenMS - singular value decomposition-based normalization; QC-RLSC - QC sample-based support vector regression normalization; VSN - variance-stabilizing normalization; MedNorm - median normalization; STD. Norm. - standard normalization; Original – no normalization; RUV-random - removal of unwanted variation-random normalization

**Supplementary Table S3 AUC**, Log2 FC, and T-tests for univariate biomarker analysis.

| No. | Name | AUC | T-tests | Log2 FC |
| --- | --- | --- | --- | --- |
| 1 | Lauroylcarnitine | 1.0 | 2.8513E-24 | -0.22012 |
| 2 | 13-HODE | 0.98633 | 1.8694000000000001E^-19^ | -0.36377 |
| 3 | 13-HOTrE | 0.98096 | 1.0282E^-15^ | -0.28698 |
| 4 | h_14_19_norandrosterone | 0.94336 | 1.2087E^-15^ | -0.40398 |
| 5 | vitamine A acetate | 0.92871 | 1.391E^-10^ | -0.22887 |
| 6 | gamma-Glutamylleucine | 0.91113 | 1.6072E^-10^ | -0.30581 |
| 7 | CEL - carboxyethyl lysine | 0.84375 | 7.0419E^-6^ | -0.25262 |
